# Extended Renal Artery Denervation Is Associated with Artery Wall Lesions and Acute Systemic and Pulmonary Hemodynamic Changes: A Sham-Controlled Experimental Study

**DOI:** 10.1155/2020/8859663

**Published:** 2020-10-28

**Authors:** Aleksandr D. Vakhrushev, Heber Ivan Condori Leandro, Natalia S. Goncharova, Lev E. Korobchenko, Lubov B. Mitrofanova, Dmitry S. Lebedev, Evgeny N. Mikhaylov

**Affiliations:** ^1^Neuromodulation Lab and Arrhythmia Department, Almazov National Medical Research Centre, Saint Petersburg, Russia; ^2^Non-Coronary Heart Diseases Department, Almazov National Medical Research Centre, Saint Petersburg, Russia; ^3^Pavlov First Saint Petersburg Medical University, Saint Petersburg, Russia; ^4^Pathology Department, Almazov National Medical Research Centre, Saint Petersburg, Russia; ^5^Department of Bioengineering Systems, Saint Petersburg Electrotechnical University “LETI”, Saint Petersburg, Russia

## Abstract

**Objectives:**

We sought to assess acute changes in systemic and pulmonary hemodynamics and microscopic artery lesions following extended renal artery denervation (RDN).

**Background:**

RDN has been proposed to reduce sympathetic nervous system hyperactivation. Although the effects of RDN on systemic circulation and overall sympathetic activity have been studied, data on the impact of RDN on pulmonary hemodynamics is lacking.

**Methods:**

The study comprised 13 normotensive Landrace pigs. After randomization, 7 animals were allocated to the group of bilateral RDN and 6 animals to the group of a sham procedure (SHAM). Hemodynamic measures, cannulation, and balloon-based occlusion of the renal arteries were performed in both groups. In the RDN group, radiofrequency ablation was performed in all available arteries and their segments. An autopsy study of the renal arteries was carried out in both groups.

**Results:**

The analysis was performed on 12 pigs (6 in either group) since pulmonary thromboembolism occurred in one case. A statistically significant drop in the mean diastolic pulmonary artery pressure (PAP) was detected in the RDN group when compared with the SHAM group (change by 13.0 ± 4.4 and 10.0 ± 3.0 mmHg, correspondingly; *P* = 0.04). In 5 out of 6 pigs in the RDN group, a significant decrease in systemic systolic blood pressure was found, when compared with baseline (98.8 ± 17.8 vs. 90.2 ± 12.6 mmHg, *P* = 0.04), and a lower mean pulmonary vascular resistance (PVR) (291.0 ± 77.4 vs. 228.5 ± 63.8 dyn∗sec∗cm^−5^, *P* = 0.03) after ablation was found. Artery dissections were found in both groups, with prevalence in animals after RDN.

**Conclusions:**

Extensive RDN leads to a rapid and significant decrease in PAP. In the majority of cases, RDN is associated with an acute lowering of systolic blood pressure and PVR. Extended RDN is associated with artery wall lesions and thrombus formation underdiagnosed by angiography.

## 1. Introduction

The autonomic nervous system is involved in the pathogenesis of cardiovascular diseases, including hypertension. In conditions such as hypertension, pulmonary arterial hypertension (PAH), coronary artery disease, chronic heart failure, excessive activation of sympathetic impulsation, and a decrease in parasympathetic activity have been noted. There is evidence that sympathetic hyperactivation makes a significant contribution to the development of both systemic hypertension and pulmonary hypertension [1].

Renal artery denervation (RDN) has been proposed to reduce sympathetic hyperactivation. Although the results of the Symplicity HTN-3 trial were neutral [2], this method has been shown effective in several newer studies [3–5].

The impact of RDN on systemic circulation and on overall sympathetic activity has been extensively studied. However, data on the effects of RDN on pulmonary hemodynamics is lacking. Thus, a few preclinical studies in small animals have evaluated right ventricle (RV) remodeling and pulmonary vascular effects of RDN [6, 7]. Importantly, these experimental studies have implemented no specialized instrumentation for RDN and may not be reproducible in larger animals or humans. Currently, there are technologies allowing radiofrequency (RF) ablation not only in the renal artery (RA) trunk but also in distal branches (extended RDN). There have been several studies analyzing acute and chronic effects of extended RDN on systemic hemodynamics [4, 8]; the acute effects of extended denervation of the RA on pulmonary hemodynamics have not been reported.

The aim of our study was to assess acute changes in systemic and pulmonary hemodynamics following extended RDN and possible associations of the ablation extent with hemodynamic changes and histologically verified artery wall damage.

## 2. Methods

The experiment was conducted on thirteen normotensive breed Landrace pigs (the mean weight 33.4 ± 2.46 kg, age 3-4 months). All procedures and protocols were reviewed and approved by the Institutional Animal Care and Use Committee (IACUC) (protocol 19-12PZ#V1). After randomization, seven animals were allocated to the group of bilateral RDN, and six animals were allocated to the group of a sham procedure (SHAM). All procedures were performed in an experimental operating room equipped with a fluoroscopic C-arm (BV Endura, Philips, Netherlands).

The animals were sedated by intramuscular injection of 1.5 ml Zoletil 100 (Virbac, Carros, France); then, the outer ear vein was cannulated, and the animals were intubated for mechanical ventilation. Ventilation was performed using the anesthetic respiratory apparatus WATO EX-35 (Shenzhen Mindray Bio-Medical Electronics Co., Ltd, China) with the following parameters: FiO_2_ 0.3, tidal volume 10 ml/kg, peak end-expiratory pressure (PEEP) 6 cm H_2_O. Anesthesia was maintained by ventilation with 1% isoflurane (Baxter Healthcare Corp., Puerto Rico). Circulating blood volume support was carried out by continuous saline and Gelofusine (B. Braun, Germany) infusion at a rate of 10 ml/kg/h each.

A longitudinal 5 cm incision was performed in the right paratracheal region; after which the right jugular vein, right common carotid artery, and the right n. vagus were isolated. A 7 F vascular hemostatic sheath (Avanti, Cordis, USA) was inserted into the right jugular vein; the 8 F 62 cm length multipurpose Preface sheath (Biosense Webster, USA) was introduced through the external carotid artery and placed in the abdominal aorta under fluoroscopic guidance. After vascular access, a solution of heparin sodium at a dose of 300 IU/kg/h was administered intravenously.

Continuous registration of invasive blood pressure (BP) was performed. The core body temperature was kept at 37°C with a thermostatically controlled blanket (WarmTouch™, Medtronic, MN, USA).

### 2.1. Renal Artery Cannulation, Stimulation, and Denervation Procedure

A 6F pigtail catheter (Cordis, Johnson and Johnson, USA) was placed in the abdominal aorta, and RA angiography was performed using 15 ml contrast media (Optiray 300, Guerbet, France).

According to previous reports, electrical high-frequency stimulation through the RA wall was suggested to identify sites with sympathetic response before RF ablation [9, 10]. The absence of stimulation-elicited BP response was considered as a potential endpoint for denervation procedures. In order to assess the possible BP reactions to RA electrical stimulation, the angiographic catheter was replaced with a 4 mm tip steerable electrophysiological catheter (Celsius, Biosense Webster, USA), which was introduced into the RA. The catheter was connected to the electrophysiological system Elcart (Electropulse, Tomsk, Russia). Electrical high-frequency 30 Hz (1 ms, 15 mA output) stimulation inside the RA was performed from at least 20 points (10 points per side). Stimulation trains were of 20 s length. Before, during, and after RA stimulation BP tracings were registered and stored.

After electrical stimulation, the catheter was exchanged with the 4 or 5 mm Vessix balloon ablation catheter, depending on the angiographic artery diameter. The balloon catheter was introduced over the PT2 guidewire 0.014in × 185 cm (Boston Scientific, USA). A solution of isosorbide dinitrate 0.1% (Isoket, EVER Pharma, Germany) was injected into the RA at a dose of 50 *μ*g. Balloon inflation was performed with diluted contrast media at 1-2 atm pressure until complete RA occlusion, as confirmed by contrast injection into the Preface sheath.

In the RDN group, ablation was performed bilaterally in all available RA branches and segments. Each RF application was 30 s in duration, temperature 65-68°C, and automatically adjusted power 0.5-1 Watt. After RDN, electrical stimulation of RAs was repeated according to the method described above.

In the SHAM group, the procedure was performed using the same protocol, including angiography, RA stimulation, nitrate injection, and the number and duration of balloon inflations. However, no RF energy was delivered.

**Figure 1:**
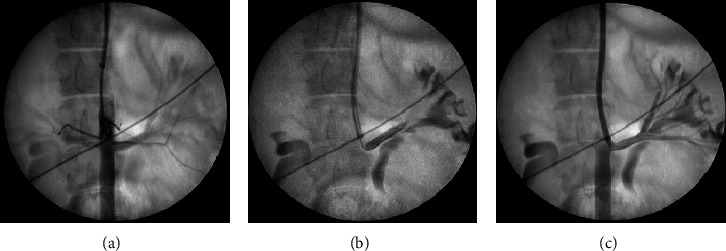
Ablation of the left RA: pig no. 3. (a) RA angiography using a pigtail catheter. (b) Inflation of a RF balloon with complete obstruction of the left main RA. (c) Repeat angiography of the RA after ablation. A slight RA lumen narrowing can be seen. RA: renal artery; RF: radiofrequency.

In both groups, selective RA angiography was performed at the end of the procedure in order to visualize potential changes of arterial lumen; all alterations were recorded (Figure 1).

### 2.2. Hemodynamic Measures

The baseline hemodynamic parameters were considered after the placement of all vascular sheaths and just before RA cannulation, and initially stable hemodynamic was noted in all animals. A Swan Ganz catheter, 6 F (B. Braun, Germany), was inserted into the distal part of the pulmonary artery (PA) through the right jugular vein under fluoroscopic control, and the pulmonary artery wedge pressure (PWP) was measured. Pressures in the PA, RV, and right atrium were measured. Arterial blood samples were taken from the abdominal aorta; venous blood samples were taken from the PA for evaluation of SatO2. Blood tests were carried out using the i-STAT portable analyzer (Abbott Laboratories, USA). Cardiac output (CO) was calculated using the Fick equation. Pulmonary vascular resistance (PVR) and systemic vascular resistance (SVR) were calculated with an established formula. Following renal artery denervation in the RDN group and balloon inflations in the SHAM group, repeated measurements were performed after a 15-minute interval.

### 2.3. Autopsy Study

At the end of the experiment, the animals were euthanized by intravenous injection of a potassium chloride solution (OZON, Russian Federation) at a dose of 800 mg. After the onset of biological death, the kidneys and abdominal aorta with the RA were excised and fixed in a solution of 10% buffered formalin as a single unit for further macroscopic and histological examinations. Immunohistochemical methods were applied to the RA specimens in both animal groups: in the RDN group, immunolabeling was performed on the specimens containing ablation sites, and in the control group on the random specimens. Immunolabeling was performed with antibodies to tyrosine hydroxylase (TH; antigen of adrenergic receptors of the sympathetic nervous system; Abcam, UK). Morphometric analysis was carried out using the image analyzer Leica Application Suite V 4.5.0 and Leica Scope (Germany). TH expression was scored as follows: 1 point—weak expression, 2—moderate expression, 3—mild expression, and 4—extensive expression.

### 2.4. Statistical Analysis

Continuous variables were presented as mean ± SD and median with interquartile ranges (IQR), where appropriate; categorical variables were presented as percentages. Group comparisons were performed using the Mann–Whitney *U* test. Comparisons within the groups were performed using Wilcoxon's test for dependent samples. Correlations were analyzed with Pearson's test. Graphical representations were made using box and whisker plots showing a mean, mean ± standard error, and mean ± 1.96∗SE. A two-sided *P* < 0.05 was considered statistically significant. The analysis was performed using the Statistica 12.0 (StatSoft Inc., Tulsa, Oklahoma, USA).

## 3. Results

The mean weight of the animals was slightly higher in the RDN group than in the controls (31.8 ± 2.8 vs. 34.8 ± 0.6 kg, correspondingly; *P* = 0.07). The total number of RF ablation points in the RDN group was 124 (20.3 ± 2.9 per animal); the range of ablation temperature was 66.7-68.3°C. Angiographic characteristics of RA and the number of RF ablation points in each artery are presented in Table 1.

### 3.1. Hemodynamic Parameters before and after Renal Denervation

Hemodynamic data obtained from one animal in the RDN group was withdrawn from the analysis, since massive left PA embolism with hemodynamic instability was diagnosed during repeat catheterization.

Therefore, hemodynamic data analysis was performed on 12 animals, 6 in either group. Changes in hemodynamic parameters in the RDN and SHAM groups are presented in Table 2.

When hemodynamic parameters were compared between the groups after the procedures (RDN or sham, according to allocation), there were significant differences in the following parameters: systolic blood pressure (sBP), mean arterial pressure (MAP), and systolic right ventricle (sRV) pressure (Table 2).

**Figure 2:**
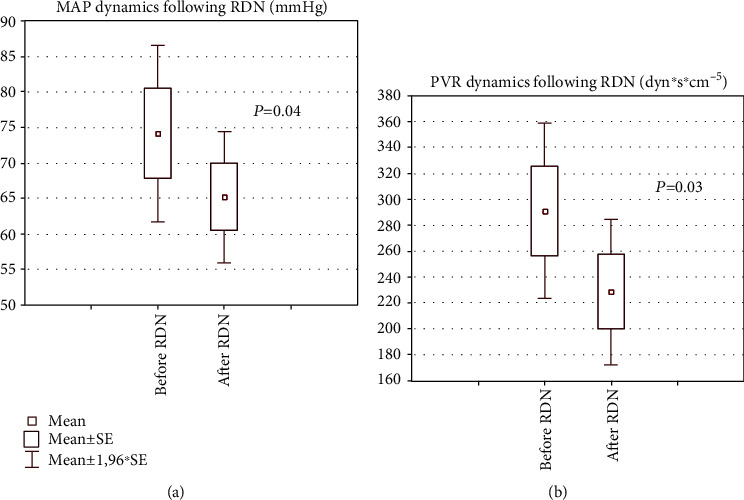
Hemodynamic parameters before and after RDN in five animals with a decrease in MAP values. (a) Mean arterial pressure before and after ablation. (b) PVR before and after ablation. RDN: renal artery denervation; PVR: pulmonary vascular resistance.

Additionally, in the RDN group, when hemodynamic parameters were compared before and after ablation, a statistically significant drop in diastolic pulmonary pressure (dPAP) was identified (13.0 ± 4.4 vs. 10.0 ± 3.0 mmHg; *P* = 0.04) (Table 2). Five out of six pigs in the RDN group showed a significant decrease in MAP. When these five animals were analyzed separately, statistically significant changes were revealed after RDN in the following parameters: a decrease in the mean sBP, when compared with the baseline values (98.8 ± 17.8 vs. 90.2 ± 12.6 mmHg, *P* = 0.04), and a decrease in MAP (74.2 ± 14.2 vs. 65.2 ± 10.6 mmHg, *P* = 0.04) and in mean PVR (291.0 ± 77.4 vs. 228.5 ± 63.8 dyn∗sec∗cm^−5^, *P* = 0.03) (see Figure 2). The sham procedure did not result in any hemodynamic change.

The total average volume of blood loss did not differ between both groups and ranged from 30 to 40 ml.

### 3.2. Electrical Stimulation of the Renal Arteries

There was no detectable influence of RA stimulation on BP or heart rate (HR) during stimulation and within 5 minutes after stimulation. The mean sBP before and immediately after electrical stimulation was 104.0 ± 14.2 mmHg vs. 103.7 ± 12.6 mmHg and diastolic BP (dBP) 67.0 ± 6.4 mmHg vs. 67.3 ± 7.2 mmHg (*P* > 0.05). The mean HR was 107.7 ± 10.5 bpm and 108.3 ± 10.3 bpm, respectively, *P* > 0.05. No BP reaction to RA stimulation was noted after RDN.

### 3.3. Angiographic Assessment of the Renal Arteries after Denervation

Local narrowing of the RA lumen was detected in 3 out of 7 pigs after RDN, and the narrowing was partly released after additional selective nitrate injection. According to their transient appearance and pharmacological response, these changes fulfilled the criteria of artery spasm. In the SHAM group, after balloon inflation, local artery changes were not detected. In total, angiographically detected RA lumen changes were detected in 23% of cases and were all attributed to radiofrequency ablation.

### 3.4. Autopsy Study

Gross anatomical evaluation of the RA showed a greater number of lesions in the RDN group compared to the SHAM group. In total, in the RDN group, twenty-three artery intimal dissections were found (3.3 ± 1.4 per animal; 0.5 ± 0.6 per artery: in trunk 0.4 ± 0.6 and in branches 0.6 ± 0.6, *P* > 0.05). Thin thrombotic masses attached to the RA wall were identified on 9 sections (1.28 ± 1.38 per animal). One case of hemorrhage in the kidney parenchyma and one parietal hematoma of the RA were identified. There was one case of total thrombosis of the left main PA.

Following RDN, in two of three cases with suspected artery spasm, dissections of the RAs were found on histological preparations at the anticipated sites of artery lumen narrowing.

There were lesions of the RA detected in 6 SHAM group animals: the mean number of artery wall lesions was lower than in the RDN group: 5 dissections (0.83 ± 0.75 per animal; *P* < 0.05) and 3 blood clots (0.5 ± 0.84 per animal; *P* < 0.05).

A positive correlation was found between the number of RF ablation points and the number of dissections (*R* = 0.84; *P* < 0.05), as well as between the number of RF ablation points and the number of blood clots (*R* = 0.88; *P* < 0.05).

**Figure 3:**
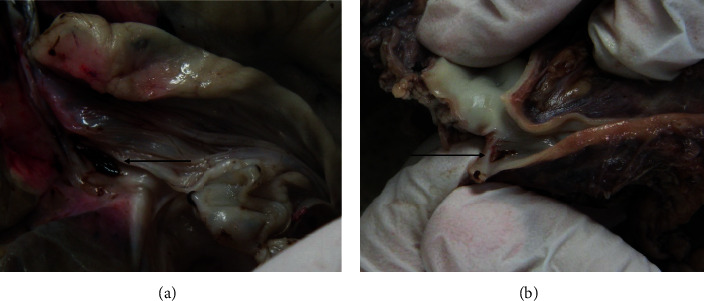
Pig #4. RA gross anatomical evaluation after RDN. (a) RA thrombosis. (b) RA dissection. RA: renal artery; RDN: renal artery denervation.

**Figure 4:**
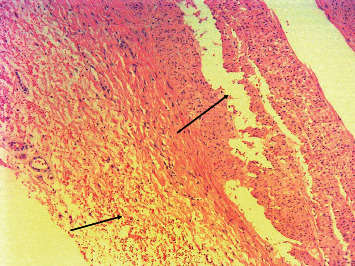
Pig #4. The arrows indicate renal artery wall dissection and hemorrhagic impregnation after RF ablation. Hematoxylin-eosin, 100x.

The shape of artery dissections was different between groups: pinpoint-like dissections in the SHAM group and larger dissections in the RDN group, with a length up to 5 mm. In the RDN group, blood clots were found in the area of the distal branches of the RA, which completely occluded the vessel lumen in one case. An example of macroscopically visible dissection and blood clotting after RDN is presented in Figure 3; a RA wall dissection detected under microscopy is shown in Figure 4.

The expression of TH was determined in both groups in all evaluated nerve fibers, in 100% of cells. The level of TH expression ranged from 3 to 4 points, and there was no difference between the groups.

## 4. Discussion

The main finding of our study is that we have identified the potential impact of extended RDN on pulmonary hemodynamics, the effect beyond systemic blood pressure reduction. In animals with a significant BP lowering after RDN, a statistically significant decrease in PVR, but not SVR, was found.

The other interesting finding is a high number of both, RF-induced and cannulation-related RA wall damage, detected on autopsy study. The majority of these lesions are not usually seen on angiography. Moreover, angiographically detected local artery narrowing following radiofrequency application can be a sign of RA dissection. The reversibility of artery narrowing after nitrate injection does not rule out the presence of microdissection.

### 4.1. Acute Blood Pressure Dynamics after Denervation

RDN is used as a method for the management of resistant systemic hypertension. The long-term clinical effects of RDN on BP have been shown in earlier and recent studies, and the major effects of RDN have been detected after several weeks following the procedure [3, 5, 11]. The acute impact of RDN on BP has been described in a number of clinical and experimental studies and is mainly explained by the change in sympathetic nervous tone after RA nerve ablation [12–14].

We suggest that when the procedure is being performed under general anesthesia the acute effects might be different from those obtained when it is conducted under light sedation. In the clinical settings, during RF energy delivery, many patients describe severe lumbar pain. It is believed that the development of pain is associated with the heating of vascular adventitia [15]. To reduce the pain in clinical practice, different approaches are used to anesthetize patients, for example, administering opioids at least 2 minutes before RF exposure and conducting combined anesthesia [16]. General anesthesia in experimental studies allows standardizing the conditions and possible hemodynamic changes after the intervention.

In our study, all procedures were performed under the same conditions, namely, all animals were of about the same age and similar morphometric indicators, and general anesthesia with intubation was performed. The difference between groups was the only RF application.

We have found a significant decrease in BP in only five out of six animals, which might be explained by the nonfully reproducible extent of catheter-based sympathetic denervation, which depends on the individual nerve distribution and artery anatomy.

There was a difference in the level of CO between the RDN and SHAM groups. This fact can be explained by a difference in the mean animals' weight in two groups.

### 4.2. Pulmonary Vascular Resistance Decrease after Denervation

The acute effect of RDN on PVR has been identified in 5 out of 6 pigs and in none of the animals from the SHAM group.

Our findings regarding the impact of RDN on pulmonary vasculature are in line with a previous report demonstrating the effects of denervation on pulmonary vascular and RV wall remodeling in chronic experiments with induced PAH [7]. In the latter study, the authors identified that PAP and the RV pressures with induced PAH were lower in animals with RDN.

### 4.3. Renal Artery Stimulation

Recently, RA electrical stimulation has been tested as an approach to BP elevation and in defining an endpoint of RDFтвN procedures [9, 10, 17]. Indeed, detecting sites within the RA that correspond to the most extensive innervation is desirable. Theoretically, electrical stimulation at those sites might elicit a sympathetic response and subsequent BP elevation. On the other hand, effective ablation of the site would have resulted in the nonreproducibility of the elicited reactions. We cannot exclude the difference in the stimulation protocols between our study and previous studies. However, a very similar stimulation approach protocol was affective in provoking autonomic reactions when used in other large vessels [18]. We suggest that pain perception during RA stimulation might be responsible for some BP elevation. In our study, the procedure was conducted under deep sedation and analgesia, precluding pain feeling. According to our findings, RA high-frequency stimulation does not elicit BP rise before and after RDN.

The absence of pain feeling under general anesthesia alleviates the possible bias of pain-associated BP elevation and effects of ablation-related pain on the measured parameters. On the other hand, acute BP and pulmonary hemodynamic reactions following extended RDN under deep anesthesia may reflect the real impact of the procedure on hemodynamics.

### 4.4. Autopsy Data

Damage to the vascular wall and/or renal parenchyma was detected in 12 (92%) animals. Although artery wall dissection was found in both groups of experimental animals, RDN was associated with more dissections, than in the SHAM group.

Previous studies compared the safety of single-electrode and multielectrode RDN systems [19]. The authors have shown that damage to both the walls of the RA and renal nerves was more prominent in the group where a multielectrode RDN system was used. However, none of the two systems led to the complete destruction of the renal nerves. We suggest that remarkable damage to the RA in our study can be associated with the use of the balloon ablation system developed for application in humans. Therefore, we cannot exclude the overstretching of the RA, which, in combination with a RF application, predispose to damage.

We have found no difference in the expression of TH between the animal groups. This finding is in line with a previous report showing that no significant TH expression decrease can be found shortly after RDN, and this might be explained by a delayed axonal degeneration in the necrotic nerves [20]. In the latter study, the authors stated that the level of TH expression cannot reflect the effectiveness of RDN in the acute phase.

### 4.5. Study Limitations

We evaluated the acute cardiovascular effects of extended RDN and did not intend to assess long-term effects. The remote effects should be addressed in a chronic experimental study.

Another limitation is the limited sample size. However, the study was exploratory and showed the effects of RDN on PVR in the majority of experimental animals. Randomized study design with a SHAM group and the conduction of the procedure under standardized conditions make the conclusions more solid.

The experiments were conducted on normotensive animals without systemic or pulmonary hypertension modeling. Different extent of effects might be expected in animals with induced hypertension or PAH.

## 5. Conclusions

In normotensive swine, extensive RDN leads to a rapid and significant decrease in BP and PVR in the majority of cases. At the same time, no significant dynamics in SVR can be detected. Extended RDN is frequently associated with artery wall dissection and thrombus formation, which seem underdiagnosed by angiography. The results of our experimental study provide a rationale for further investigation of extended RDN effects on pulmonary circulation in chronic PAH models.

## Figures and Tables

**Table 1:** Angiographic characteristics of renal arteries and number of RF ablation points in each animal.

Animal #	Group	Main RA diameter (mm)	RA branch diameter (mm)	Number of ablation points
1	RDN	4.1	2.9	22
2	RDN	3.7	2.8	23
3	SHAM	4.0	2.9	—
4	RDN	3.3	2.3	16
5	SHAM	3.7	2.6	—
6	RDN	3.6	3.3	22
7	RDN	3.9	2.8	22
8	SHAM	3.9	2.7	—
9	RDN	4.9	3.8	23
10	SHAM	3.6	2.4	—
11	SHAM	4.2	2.8	—
12	RDN	4.2	2.5	18
13	SHAM	3.9	2.8	—
	Total	3.9 ± 0.4	2.8 ± 0.4	20.3 ± 2.9

**Table 2:** Hemodynamic changes in the RDN and SHAM groups.

	RDN group (*N* = 6)		SHAM group (*N* = 6)		*P*, between groups, when parameters were analyzed after procedures	A subgroup of RDN animals with MAP drop: delta in parameters after ablation, median [IQR], *N* = 5	*P*, five RDN cases with MAP drop, comparison of parameters before and after ablation
	Before RDN, mean ± SD	After RDN, mean ± SD	Before sham, mean ± SD	After sham, mean ± SD			
HR (min^−1^)	116.7 ± 15.1	118.2 ± 21.8	95.8 ± 11.7	102.2 ± 19.1	0.24	3 [-2; 12]	0.34
sBP (mmHg)	97 ± 16.5	90.8 ± 11.4	88.3 ± 7.5	93.2 ± 8	0.03	-6 [-14; -6]	0.04
dBP (mmHg)	60.3 ± 11.6	56 ± 8.2	54.5 ± 6.4	60.2 ± 6	0.05	-7 [-9; -4]	0.14
MAP (mmHg)	72.7 ± 13.2	66 ± 9.6	65.7 ± 6.4	71.2 ± 6.6	0.04	-7 [-10; -6]	0.04
sPAP (mmHg)	19.5 ± 4.3	18.3 ± 5.2	15.7 ± 4	15 ± 3.4	0.24	-1 [-1; 0]	0.27
dPAP (mmHg)	13 ± 4.4	10 ± 3	11.8 ± 3.8	10.7 ± 2.4	0.82	-2 [-5; -1]	0.06
mPAP (mmHg)	15.1 ± 4.1	13.3 ± 2.9	13 ± 3.9	12.2 ± 2.9	0.48	-1 [-4; 0]	0.11
sRV (mmHg)	18.5 ± 6.6	19.3 ± 5.2	13.8 ± 4.3	13.3 ± 3.1	0.04	-1 [-3; 3]	0.89
dRV (mmHg)	5 ± 2.6	4.5 ± 2.6	6.5 ± 3.3	6 ± 1.3	0.48	0 [-1; 0]	0.18
mRV (mmHg)	9.3 ± 2.8	9.3 ± 2.2	8.8 ± 3.7	8.2 ± 1.7	0.31	-1 [-1; -1]	0.22
PWP (mmHg)	4.7 ± 1.8	4 ± 1.1	5 ± 2.4	4.7 ± 1.9	0.39	-1 [-2; 0]	0.47
RAP (mmHg)	3.8 ± 2.7	2 ± 1.7	4.4 ± 2.4	3.2 ± 1.6	0.24	-3 [-3; -2]	0.07
SVR (dyn∗sec∗cm^−5^)	1747.7 ± 726.1	1544.9 ± 725.8	1113.4 ± 520.3	1193.1 ± 589.7	0.24	-252.9 [-591.7; 96.0]	0.22
PVR (dyn∗sec∗cm^−5^)	261.9 ± 99.3^†^	215.1 ± 65.9	136.9 ± 58.5^†^	133.7 ± 86.3	0.06	-88.0 [-92.4; -36.5]	0.03
CO (l/min)	3.6 ± 1.5	3.7 ± 1.2	5 ± 1.8	5.4 ± 2.3	0.24	0.2 [-0.3; 0.5]	0.68

HR: heart rate; sBP: systolic blood pressure; dBP: diastolic blood pressure; MAP: mean arterial pressure; sPAP: systolic pulmonary artery pressure; dPAP: diastolic pulmonary artery pressure; mPAP: medium pulmonary artery pressure; sRV: systolic right ventricle pressure; dRV: diastolic right ventricle pressure; mRV: medium right ventricle pressure; PWP: pulmonary wedge pressure; RAP: right atrial pressure; SVR: systemic vascular resistance; PVR: pulmonary vascular resistance; CO: cardiac output. ^†^*P* = 0.03 in baseline parameters between the groups.

## Data Availability

The data used to support the findings of this study are available from the corresponding author upon reasonable request.

## References

[1] Vaillancourt M., Chia P., Sarji S. (2017). Autonomic nervous system involvement in pulmonary arterial hypertension. *Respiratory Research*.

[2] Bhatt D. L., Kandzari D. E., O'Neill W. W. (2014). A controlled trial of renal denervation for resistant hypertension. *The New England Journal of Medicine*.

[3] Mahfoud F., Böhm M., Schmieder R. (2019). Effects of renal denervation on kidney function and long-term outcomes: 3-year follow-up from the Global SYMPLICITY Registry. *European Heart Journal*.

[4] Böhm M., Kario K., Kandzari D. E. (2020). Efficacy of catheter-based renal denervation in the absence of antihypertensive medications (SPYRAL HTN-OFF MED Pivotal): a multicentre, randomised, sham-controlled trial. *Lancet*.

[5] Kandzari D. E., Böhm M., Mahfoud F. (2018). Effect of renal denervation on blood pressure in the presence of antihypertensive drugs: 6-month efficacy and safety results from the SPYRAL HTN-ON MED proof-of-concept randomised trial. *Lancet*.

[6] da Silva Gonçalves Bos D., Happé C., Schalij I. (2017). Renal denervation reduces pulmonary vascular remodeling and right ventricular diastolic stiffness in experimental pulmonary hypertension. *JACC Basic Transl Sci.*.

[7] Qingyan Z., Xuejun J., Yanhong T. (2015). Efectos beneficiosos de la simpatectomía renal sobre el remodelado vascular pulmonar en la hipertensión arterial primaria experimental. *Revista Española de Cardiología*.

[8] Pekarskiy S. E., Baev A. E., Mordovin V. F. (2017). Denervation of the distal renal arterial branches vs. conventional main renal artery treatment: a randomized controlled trial for treatment of resistant hypertension. *Journal of Hypertension*.

[9] Madhavan M., Desimone C. V., Ebrille E. (2014). Transvenous stimulation of the renal sympathetic nerves increases systemic blood pressure: a potential new treatment option for neurocardiogenic syncope. *Journal of Cardiovascular Electrophysiology*.

[10] Chinushi M., Izumi D., Iijima K. (2013). Blood pressure and autonomic responses to electrical stimulation of the renal arterial nerves before and after ablation of the renal artery. *Hypertension*.

[11] Donazzan L., Mahfoud F., Ewen S. (2016). Effects of catheter-based renal denervation on cardiac sympathetic activity and innervation in patients with resistant hypertension. *Clinical Research in Cardiology*.

[12] Fink G. D., Phelps J. T. (2017). Can we predict the blood pressure response to renal denervation?. *Autonomic Neuroscience*.

[13] Cohen-Mazor M., Mathur P., Stanley J. R. L. (2014). Evaluation of renal nerve morphological changes and norepinephrine levels following treatment with novel bipolar radiofrequency delivery systems in a porcine model. *Journal of Hypertension*.

[14] Vogiatzakis N., Tsioufis C., Georgiopoulos G. (2017). Effect of renal sympathetic denervation on short-term blood pressure variability in resistant hypertension. *Journal of Hypertension*.

[15] Sapoval M., Azizi M., Bobrie G., Cholley B., Pagny J. Y., Plouin P. F. (2012). Endovascular renal artery denervation: why, when, and how?. *Cardiovascular and Interventional Radiology*.

[16] Pansieri M., Barnay P., Larderet E. (2013). Dénervation rénale pour HTA réfractaire sans anesthésie générale : intérêt d’un protocole MEOPA Morphine. Expérience préliminaire. *Annales de Cardiologie et d'Angéiologie*.

[17] Tsiachris D., Tsioufis C., Dimitriadis K. (2014). Electrical stimulation of the renal arterial nerves does not unmask the blindness of renal denervation procedure in swine. *International Journal of Cardiology*.

[18] Goncharova N. S., Moiseeva O. M., Condori Leandro H. I. (2020). Electrical stimulation-guided approach to pulmonary artery catheter ablation in patients with idiopathic pulmonary arterial hypertension: a pilot feasibility study with a 12-month follow-up. *BioMed Research International*.

[19] Táborský M., Richter D., Tonar Z. (2017). Early morphologic alterations in renal artery wall and renal nerves in response to catheter-based renal denervation procedure in sheep: difference between single-point and multiple-point ablation catheters. *Physiological Research*.

[20] Su E., Zhao L., Gao C. (2019). Acute changes in morphology and renal vascular relaxation function after renal denervation using temperature-controlled radiofrequency catheter. *BMC Cardiovascular Disorders*.

